# Radiation-induced malignant fibrous histiocytoma of the occipital: a case report

**DOI:** 10.1186/1477-7819-12-98

**Published:** 2014-04-17

**Authors:** Guo-Bin Zhang, Jian Li, Peng-Fei Zhang, Li-Jiang Han, Jun-Ting Zhang

**Affiliations:** 1 Department of Neurosurgery, Beijing Tiantan Hospital, Capital Medical University, Tiantan Xili 6, Chongwen District, Beijing 100050, PR China

**Keywords:** Malignant fibrous histiocytoma, Radiation induced sarcoma, Occipital

## Abstract

Malignant fibrous histiocytoma (MFH) is a rare neoplasm exhibiting a propensity for aggressive clinical behavior. Effective treatment modality is surgical resection with wide margins, but its rate of recurrence and metastasis is still high. Early detection and complete excision of the tumor is necessary. A MFH of the occipital developed in a 51-year-old woman eight years after surgery and radiation for medulloblastoma of the cerebellar vermis. The secondary neoplasm arose at the site of tumor resection within the irradiated field, and was resected. The development of sarcomas is a recognized complication of radiation therapy. The final diagnosis after the operation was MFH. Radiation-induced sarcoma (RIS) is well known, but radiation-induced MFH is relatively rare in the head and neck region, especially in the occipital. The imaging findings are not diagnosis specific, but strict follow-up within the radiation field by computerized tomography (CT) and magnetic resonance imaging (MRI) and appreciation of the expected latency period may help in providing the diagnosis of RIS.

## Background

Malignant fibrous histiocytoma (MFH) is one of the most common soft tissue sarcomas of adulthood and has been reported in various organs. The site of primary origin tends to be mainly in the extremities followed by the trunk, the head, and the neck [1]. MFH are rare tumors within the central nervous system. However, they have been reported in the brain, dura mater, cranial bones, spine and peripheral nerves [2,3]. Radiotherapy is one of the most important treatments of malignant tumors in the head and neck region, but it is known to induce major side effects such as radionecrosis and oncogenesis. MFH has been reported to occur with increased frequency in patients who have been treated with radiotherapy for malignant disease [4]. The purpose of this report is to improve the understanding of the clinical behavior, predictors of biological aggressiveness, and histological and immunohistochemical features of MFH after radiotherapy for medulloblastoma of the cerebellar vermis.

## Case presentation

### Clinical presentation

#### History and examination

**Figure 1:**
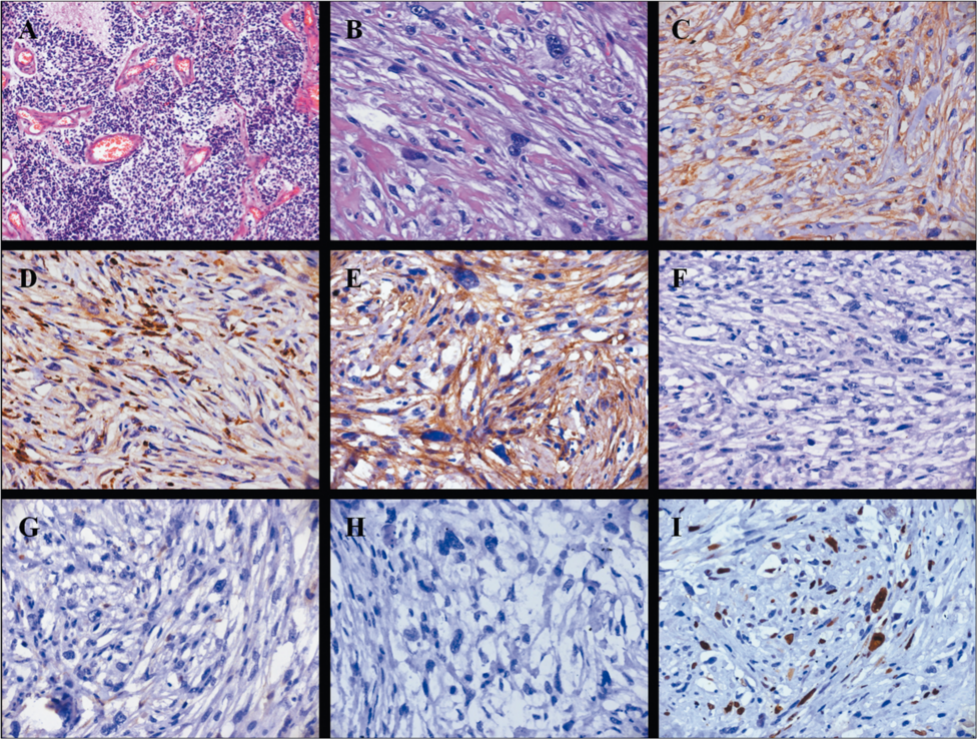
**Pathological characteristics of the patient. A)** High-power photomicrograph of the lesion, eight years previously, which was diagnosed as medulloblastoma (H & E, 200×). **B)** High-power photomicrograph showing proliferation of pleomorphic spindle-shaped cells having large irregular nuclei with hyperchromasia (H & E, 200×). **C**-**I)** Photomicrograph of tumor with immunohistochemical stain (200×). **C)** Vimentin demonstrating a strongly positive reaction in the tumor cells. **D)** CD68 demonstrating a strongly positive reaction in the tumor cells. **E)** Fibronectin (FN) demonstrating a strongly positive reaction in the tumor cells. **F)** Desmin showing negative reaction in the tumor cells. **G)** S-100 showing negative reaction in the tumor cells. **H)** Cytokeratins showing negative reaction in the tumor cells. **I)** Ki-67 (MIB-1) demonstrating positive reaction in the tumor cells. The MIB-1 labeling index is 10%.

**Figure 2:**
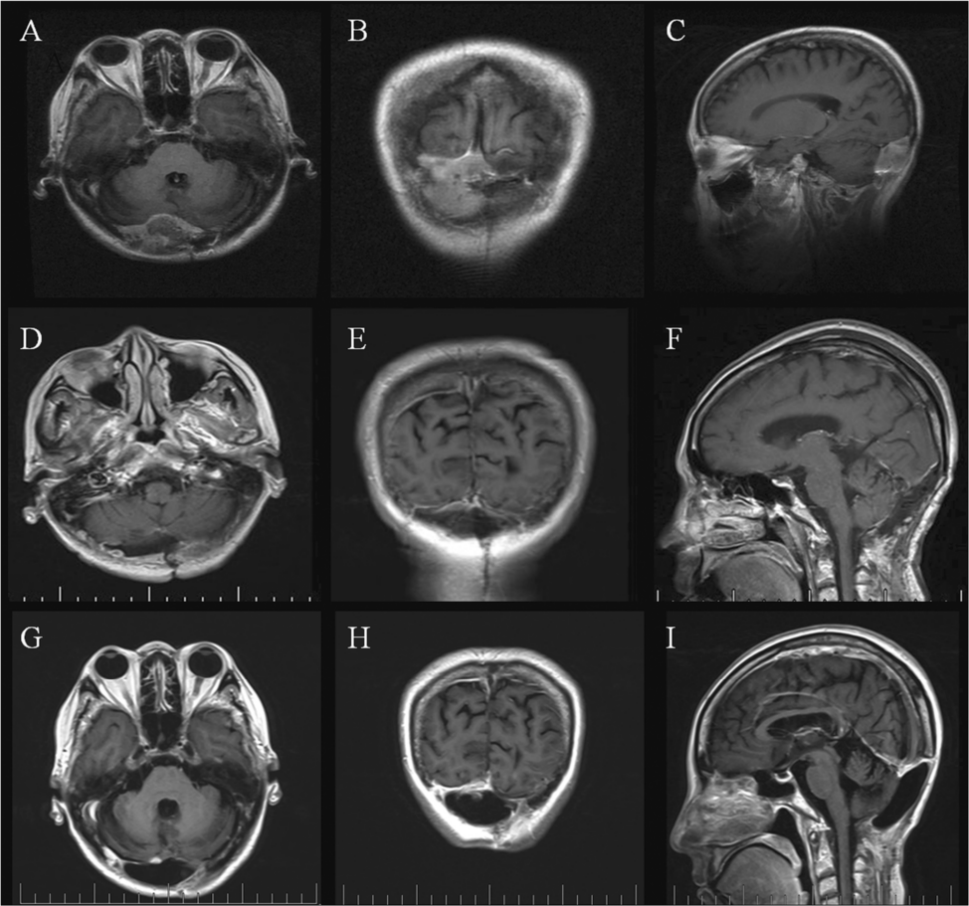
**MR images showing the MFH of the posterior cranial fossa in preoperative and postoperative follow-up. A, B, C)** MR images showing an expansive mass of the posterior cranial fossa with an irregular outline involving the right cerebellar hemispheres, posterior fossa dura, occipital bone and the torcular herophili. The signal intensity of the lesion is slightly hyperintense on the T1-weighted Gd-enhanced image. T1-weighted Gd-enhanced image showing moderate heterogeneous enhancement throughout the lesion. **D, E, F)** Postoperative early stage MR images showing no residual mass lesion. **G, H, I)** Follow-up MRI on the postoperative sixth month showing tumor non-recurrence. MR, magnetic resonance.

A 51-year-old woman was first admitted at age 43 years in July 2005. A midline suboccipital craniotomy of the cerebellar vermis was performed in our department after medulloblastoma was diagnosed (Figure 1A). Postoperative irradiation was delivered in daily doses of 2 Gy, totaling an overall dose of 60 Gy. Her five-year follow-up was completed. She returned to our hospital eight years after surgery (in July 2013), because of bilateral blurry vision for two months. Neurological examination revealed bilateral papilledema and slight cerebellar ataxia. No abnormality was found on routine hematologic examination or urinalysis. Magnetic resonance imaging (MRI) showed an expansive and heterogeneous enhanced contrast mass with an irregular outline involving the right cerebellar hemispheres, posterior fossa dura, occipital bone, and the torcular herophili measuring 5.2 × 3.2 × 3.2 cm, consistent with recurrent medulloblastoma (Figure 2A,B,C).

### Operation and pathological findings

The patient underwent a midline suboccipital craniectomy and complete resection of the tumor. Afterwards, total closure of the dura defect was completed by reconstruction using artificial dura mater to protect the brain tissue. Intraoperatively the tumor was very vascular, gritty, necrotic in some areas and corroded the occipital bone. Postoperative early stage MRI confirmed no residual mass lesion (Figure 2D,E,F). Histopathological examination revealed a MFH. Hematoxylin and eosin (H & E) stained slides revealed anaplastic proliferation of spindle-shaped cells admixed with atypical giant cells arranged in fascicular and storiform patterns (Figure 1B). These cells contained typical hyperchrome nuclei with moderate cytoplasms and mitosis. The immunohistochemical stains showed vimentin, CD68 and fibronectin (FN) expression by the tumor cells (Figure 1C,D,E). Desmin, S-100 and cytokeratins were negative (Figure 1F,G,H). The Ki-67 proliferation index was 10% (Figure 1I). Genomic DNA was isolated from peripheral blood mononuclear cells according to standard protocols. For mutation analysis of the complete coding region and the exon–intron junctions of *MLH1* and *MSH2* in patients with microsatellite instability, direct DNA sequencing after PCR amplification was applied [5,6]. Genomic mutations in *MLH1* or *MSH2* were not detected in our patient.

### Postoperative follow-up

The postoperative course was unremarkable and the patient was discharged after seven days. She rejected any radiotherapy or chemotherapy protocol in follow-up treatment. Follow-up MRI and whole-body positron emission tomography-computed tomography (PET-CT) in the postoperative sixth month demonstrated tumor non-recurrence at the same location and non-distant metastasis (Figure 2G,H,I).

## Discussion

MFH is one of the potentially devastating sarcomas in middle-aged or elderly adults, frequently arising from the proximal end of the extremities and in the retroperitoneum [7]. It is uncommon in the head and neck region, its frequency in this region being only 1% to 3% of all MFHs [8]. Clinical manifestations of intracranial MFH are various and mainly depend on the location of the tumor. Occipital MFH has no distinct clinical feature that could aid in its recognition. The diagnosis of MFH is based primarily upon excellent sampling in conjunction with a targeted panel of immunohistochemistry [9]. MFH tumor is composed of an admixture of spindle-shaped fibroblastic tumor cells and bizarre mononuclear histiocytic tumor cells arranged in a storiform pattern with some multinucleated giant cells [7]. Immuno-positivity for vimentin, CD68 and Ki-67 has been demonstrated in MFH and is helpful for diagnosis. The tumor tissue should be immune-negative for S-100 protein and cytokeratins. The differential diagnosis of MFH can be difficult because of its similarity to other sarcomas composed of spindle cells, such as spindle cell carcinoma, carcinosarcoma and fibrosarcoma. Sometimes, microscopy with routine staining does not allow distinction of this sarcoma from others, especially when the cells are pleomorphic. Although the characteristic patterns, such as the storiform and antler-like patterns, are useful, immunohistochemical staining was helpful for the diagnosis in our case.

Brieger *et al*. describe two patients with Lynch syndrome-associated MFH. Both patients carried a MSH2 germline mutation and a mutator phenotype with loss of MSH2 expression could be demonstrated in both MFH [10]. This finding further supports the potential risk of patients with Lynch syndrome to develop a MFH as a consequence of the underlying MMR gene germline mutation [10,11]. In our case, the genomic mutations in MLH1 or MSH2 were not detected, and the clinical presentation also did not confirm the diagnosis of Lynch syndrome-associated MFH.

MFH has been reported to be more common among patients who have received radiotherapy [12]. Ionized radiation is clearly known to trigger sarcoma formation [13]. The following criteria for defining a radiation induced sarcoma (RIS) have been proposed by Cahan *et al*. [14]: (1) evidence of an initial distinct malignant tumor different from the subsequent sarcoma; (2) development of the second malignant tumor in an irradiated field; (3) long interval between radiation and development of the sarcoma (a latency period of not less than five years); and (4) histological confirmation of sarcoma. The present case fulfilled all these criteria.

It is difficult to analyze the relationship between the total irradiation dose received and the incidence of RIS of the head and neck. Kirova *et al*. [15] showed that most reported cases of RIS after breast irradiation occurred after the administration of 60 to 80 Gy, with a minimal dose of 10 Gy, in standard fractions. Karlsson *et al*. [16] found that the risk of sarcoma other than angiosarcomas increased linearly with a total dose of 150 to 200 J, stabilizing at higher energies. Pitcher *et al*. [5] believe that RIS may appear following radical radiation therapy regardless of the photon energy. However, no available studies provide data on the relationship between the prescribed radiation dose and RIS. RIS typically occurs within or at the edge of the radiation field [6]. Radiation doses greater than 50 Gy cause complete devitalization and bone death, while lower doses greater than 30 Gy are associated with permanent damage to the reparative mechanisms [17]. The edge of the radiation field is, therefore, at particular risk for the development of a secondary malignancy. The lack of homogeneity of the dose at the periphery of the radiation field also results in an area that has received less radiation than the putative tumor dose. The radiation dose here may be insufficient to kill all viable cells, but may be sufficient to cause cell damage and genetic mutations. A disorganized reparative proliferation may then act as an initiating factor in tumor development.

The RIS is characterized by an aggressive behavior, with a high incidence of local recurrence and distant metastasis [18]. In our patient, the recurrent disease and distant metastasis did not occur within a few months postoperatively. The overall five-year survival of RIS ranges between 10% and 30% [18,19]. Most papers quote a poor outcome for RIS in the head and neck; for instance, Mark *et al*. [20] reported a year disease-free survival of only 8%. Wang *et al*. [21] reported that the five-year disease-free survival and five-year overall survival rates of radiation-induced MFH of the sinonasal tract were 0 and 5.9%.

The preferred treatment is surgical resection whenever the site and size of tumor allow it. Radical *en bloc* resection is required because of the aggressiveness of MFH, both locally and remotely [22]. Sabesan *et al*. [23] reported that marginal resection of MFH of the head and neck carries a local recurrence rate of up to 85%, whereas radical resection has a local recurrence risk of only 27%. Because of the technical difficulty of determining adequate surgical margins and the poor healing and wound complications associated with radiation changes, surgical management of RIS in the posterior fossa is also challenging. Radiation therapy for radiation-induced MFH is effective in some cases but not in others [24]. The effectiveness of chemotherapy is also controversial and has yet to demonstrate definitive benefit [9]. Ettinger *et al*. [25] reported excellent results using postoperative cisplatin and doxorubicin in patients with non-metastatic osteosarcoma. A recent study by Hugate *et al*. [26] reported that neoadjuvant chemotherapy (intraarterial cisplatin and intravenous doxorubicin) improved survival rates of primary high-grade extremity osteosarcoma and MFH of the bone. Kocer *et al*. [27] showed that neoadjuvant chemotherapy was an effective method for radiation-induced sternal MFH. In planning the surgical resection, the size of the tumor before chemotherapy should be considered as the initial size and surgical margins should be determined accordingly. Ko *et al*. [28] reported that exploitation of more effective chemotherapy is necessary to improve the tumor-free survival of patients with radiation-induced MFH in the head and neck region. With so few reported cases of RIS, it is difficult to establish the optimal treatment. Initial radical surgery is usually the effective treatment for achieving long-term survival because adjuvant and palliative treatments are not so effective [1].

## Conclusions

A poor prognosis can be expected because extensive removal of the MFH is not easily accomplished. Owing to its high rate of recurrence and metastasis, early detection and complete excision of the tumor is necessary. The imaging findings are not diagnosis specific, but strict follow-up within the radiation field by CT and MRI and appreciation of the expected latency period may help in providing the diagnosis of RIS.

## Consent

Written informed consent was obtained from the patient for publication of this case report and any accompanying images. A copy of the written consent is available for review by the editor-in-chief of this journal.

## Abbreviations

CT: computed tomography; H & E: hematoxylin and eosin; MFH: malignant fibrous histiocytoma; MRI: magnetic resonance imaging; RIS: radiation-induced sarcoma.

## Competing interests

The authors declare that they have no competing interests.

## Authors’ contributions

GBZ, JL and JTZ reviewed the literature and drafted the manuscript. GBZ, PFZ, LJH and JTZ were clinically responsible for the patient’s care and revised the manuscript. JL and LJH were responsible for the pathology and radiological images. All authors read and approved the final manuscript.
